# Revealing Opinions for COVID-19 Questions Using a Context Retriever, Opinion Aggregator, and Question-Answering Model: Model Development Study

**DOI:** 10.2196/22860

**Published:** 2021-03-19

**Authors:** Zhao-Hua Lu, Jade Xiaoqing Wang, Xintong Li

**Affiliations:** 1 Department of Biostatistics St. Jude Children’s Research Hospital Memphis, TN United States; 2 Department of Linguistics The Ohio State University Columbus, OH United States

**Keywords:** natural language processing, question-answering systems, language summarization, machine learning, life and medical sciences, COVID-19, public health, coronavirus literature

## Abstract

**Background:**

COVID-19 has challenged global public health because it is highly contagious and can be lethal. Numerous ongoing and recently published studies about the disease have emerged. However, the research regarding COVID-19 is largely ongoing and inconclusive.

**Objective:**

A potential way to accelerate COVID-19 research is to use existing information gleaned from research into other viruses that belong to the coronavirus family. Our objective is to develop a natural language processing method for answering factoid questions related to COVID-19 using published articles as knowledge sources.

**Methods:**

Given a question, first, a BM25-based context retriever model is implemented to select the most relevant passages from previously published articles. Second, for each selected context passage, an answer is obtained using a pretrained bidirectional encoder representations from transformers (BERT) question-answering model. Third, an opinion aggregator, which is a combination of a biterm topic model and k-means clustering, is applied to the task of aggregating all answers into several opinions.

**Results:**

We applied the proposed pipeline to extract answers, opinions, and the most frequent words related to six questions from the COVID-19 Open Research Dataset Challenge. By showing the longitudinal distributions of the opinions, we uncovered the trends of opinions and popular words in the articles published in the five time periods assessed: before 1990, 1990-1999, 2000-2009, 2010-2018, and since 2019. The changes in opinions and popular words agree with several distinct characteristics and challenges of COVID-19, including a higher risk for senior people and people with pre-existing medical conditions; high contagion and rapid transmission; and a more urgent need for screening and testing. The opinions and popular words also provide additional insights for the COVID-19–related questions.

**Conclusions:**

Compared with other methods of literature retrieval and answer generation, opinion aggregation using our method leads to more interpretable, robust, and comprehensive question-specific literature reviews. The results demonstrate the usefulness of the proposed method in answering COVID-19–related questions with main opinions and capturing the trends of research about COVID-19 and other relevant strains of coronavirus in recent years.

## Introduction

COVID-19 is an infectious disease that emerged in late 2019 and has resulted in an ongoing pandemic [1]. COVID-19 is caused by SARS-CoV-2, which belongs to a family of viruses known as coronaviruses. Coronaviruses are enveloped positive-sense single-stranded ribonucleic acid viruses. Recently discovered coronaviruses include severe acute respiratory syndrome coronavirus (SARS-CoV) [2] and Middle East respiratory syndrome coronavirus (MERS-CoV) [3]. COVID-19 has challenged global public health because it is highly contagious and can be lethal. Consequently, numerous ongoing and recently published studies have emerged in the recent literature. However, research regarding COVID-19 is largely ongoing and inconclusive.

A potential approach that could accelerate COVID-19 research is to use information from the existing research into other viruses that belong to the coronavirus family. To address the COVID-19 pandemic challenges, the US government and several leading research groups have created the COVID-19 Open Research Dataset (CORD-19) [4], which includes scholarly articles about COVID-19 and related coronaviruses. However, it would be difficult for the medical research community to keep up with the continuously growing literature. The application of natural language processing (NLP), together with relevant statistical techniques, can help to generate new insights and support the ongoing fight against COVID-19.

Our motivation is to address the two important questions raised by the CORD-19 Challenge: (1) use natural language processing to find answers to questions within, and connect insights across, the CORD-19 database in support of the ongoing COVID-19 response efforts worldwide; and (2) help the medical community keep up with the rapid increase in COVID-19 literature. Our goal is to make use of these techniques to distill information from the CORD-19 data set and generate answers and summaries for a variety of questions from different perspectives. In addition, we will demonstrate the longitudinal trends of the research related to the coronavirus family and compare the answers with our existing understanding of COVID-19 and other viruses in the coronavirus family.

## Methods

### Overview

**Figure 1:**
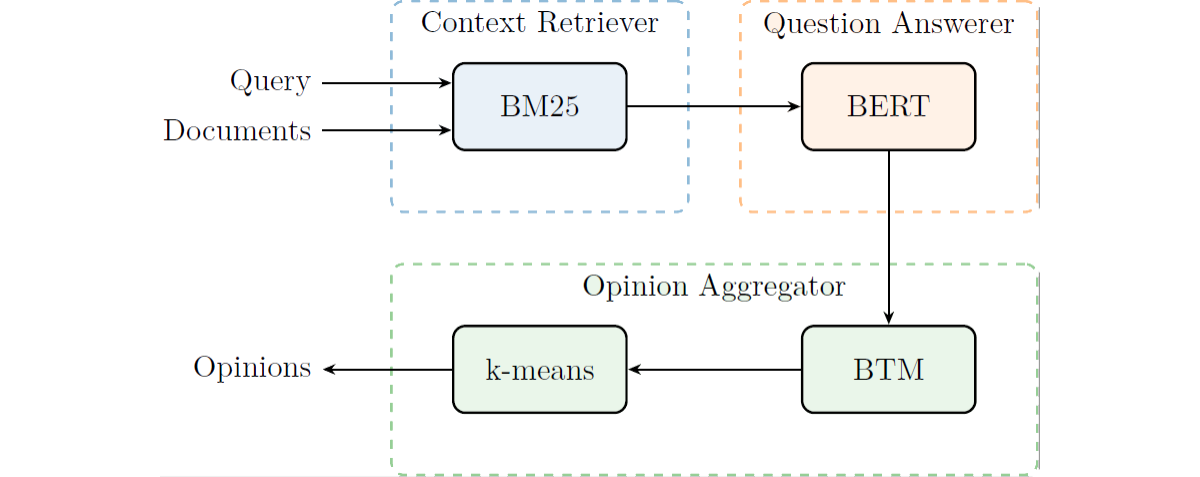
Illustration of the pipeline. BERT: bidirectional encoder representations from transformers; BTM: biterm topic model.

We propose a pipeline to automatically answer various COVID-19–related questions from the literature and aggregate the answers into several main opinions. The pipeline consists of three components as shown in Figure 1: (1) a context retriever module for finding relevant passages from the articles, (2) a question-answering model for extracting answers from a single context passage, and (3) an opinion aggregator for clustering answers into opinions. The popularity of an opinion is quantified by the number of supportive answers in the literature. We show the trends of the related research by investigating the research trends quantified by the longitudinal distributions of the opinions based on the answers to each question.

### Related Work

The release of the CORD-19 data set and the call for using NLP techniques to exploit CORD-19 have inspired considerable research effort toward building systems that can help researchers to explore valuable information related to COVID-19. Some earlier contributions, including CORD-19 Search [5], tmCOVID [6], WellAI COVID-19 Research Tool [7], and Covidex [8], focus on search engine development or identification of medical concepts. On the other hand, with the emerging requests from both the medical research community and society in general to find answers to various questions regarding COVID-19, systems that can provide reliable answers to COVID-19–related questions using the latest COVID-19 research are urgently needed.

Some very recent developments in answering systems built on top of the CORD-19 corpus include CovidQA [9], CAiRE-COVID [10], and CO-Search [11]. These methods tried to generate answers for COVID-19–related questions by locating a fraction of the documents in CORD-19 or summarizing the documents into a final answer through text generation (eg, BART [12]).

Compared to these very recent works, our research has several unique contributions. First, for a user query, our proposed system can automatically aggregate the answers from the most related articles into a few main opinions, which helps researchers to acquire clear directions. Second, the opinions are generated with popularity measurement as the number of supportive answers in the selected articles. Given that COVID-19 is being actively researched, multiple opinions accompanied by popularity measurements may answer open questions better than the definitive answers produced by the other methods. Third, our research pays more attention to data analyses and interpretations, while other research focuses on model performance evaluation. Nonetheless, we provide clear justifications for the selected components in our pipeline. Below, we describe our pipeline’s three components: a context retriever, a question-answering model, and an opinion aggregator (Figure 1).

### Context Retriever

Using a neural model to extract answers from passages of the entire literature is costly, and it is more efficient to filter the irrelevant passages out first. Chen et al [13] used term frequency–inverse document frequency (TF-IDF) first to narrow the search space so that the following steps could focus only on the relevant passages. Likewise, we use BM25 [14,15] as an enhanced version of TF-IDF to quantify the relevance of the passages deterministically and select the relevant passages. Compared to the linear term frequency score in TF-IDF, BM25 alleviates the imbalance impact of high term frequency by limiting the range of the term frequency score to represent term saturation (ie, after the term appears a sufficient number of times, a higher frequency of the term does not affect the relevance of a passage with the term). Furthermore, BM25 uses the specific document length relative to the average document length to determine the sufficient term number for saturation, resulting in a more flexible and stable context retriever. Specifically, the question and the passages are regarded as a bag of words, and they are compared through BM25 similarity. For each question, all passages are ranked according to their similarity values with the question, and only the top passages are selected.

### Question-Answering Model

Given a question and a passage, we apply the bidirectional encoder representations from transformers (BERT) [16,17] question-answering model pretrained by Huggingface [18] to each passage selected by the context retriever to predict the location and span of answer text in each passage. We chose the large, uncased version of BERT. Specifically, the question and the passage are packed into a single sequence separated by a segment embedding. Their contextual representations are computed by BERT. The start position of the answer in the passage is the word with the contextural representation that leads to the largest inner product with the start vector in the pretrained BERT question-answering model. The end position is similarly calculated using the end vector in the pretrained model. For more details, please refer to section 4.2 in [16].

### Opinion Aggregator

To better understand the structures and opinions of the selected answers, we propose an efficient way to aggregate and summarize the answers for a question into a few main opinions through a modified topic model. It was shown that the topical feature vectors produced by the topic models are informative in classification tasks [19,20]. We propose to use a topic model to generate the feature vectors for each answer. However, the number of topics is hard to determine in advance and tuning the number of topics in a topic model is costly because the convergence of a topic model may require considerable iterations. Instead, we propose to aggregate the answers by clustering them according to their topical feature vectors by k-means clustering due to its efficiency in clustering. Each cluster represents an opinion for a question. The meaning of an opinion is represented by the answers in the corresponding cluster.

In our preliminary experiments, we found that the lengths of the answers are substantially different. The traditional topic models—latent Dirichlet allocation [20] and probabilistic latent semantic analysis—are known to suffer from the sparsity problem when the answers are short [21]. Hence, we use the biterm topic model (BTM) [19,22] as an enhanced topic model for distilling the topics that underlie the answers. Rather than using single words, BTM is based on all possible unordered word pairs (ie, biterms) so as to alleviate the sparsity problem caused by applying traditional topic models to short texts. Specifically, we consider all the answers as a mixture of topics, where each biterm is drawn from a specific topic independently. The probability that a biterm is drawn from a specific topic is further captured by the likelihood that both words in the biterm are drawn from the topic.

The estimation and inference based on the BTM (ie, the affiliation of the answers to the topics) can be obtained by Gibbs sampling based on posterior distributions [19]. By applying BTM, a topical feature vector is obtained for each answer. The dimension we used is 40. The topical feature vectors for all answers are clustered by the k-means algorithm, where the best number of the clusters k* is selected using the Silhouette coefficient [23,24]. Each opinion is named by the supportive answer (in the cluster) that is closest to the centroid of a cluster. Its popularity is represented by the number of answers belonging to that cluster. For each opinion, the word frequencies of the corresponding answers are counted. The most frequent words are used together with the opinion name for interpretation.

### Proposed Pipeline

#### Data Set

We conducted experiments on the CORD-19 data set. The data set contains 47,000 articles along with their titles, abstracts, publication dates, and other metadata. Throughout this paper, we use version 2020-04-03 of the data set. In total, six questions from the CORD-19 data set are used as a demonstration. The proposed pipeline is applicable to general questions.

#### Context Retriever

We concatenated the title and the abstract of an article as a single context passage for calculating the similarity in the context retrieving step. Note that the Natural Language Toolkit’ stop words [25] are not included in both the context passages and the questions. When calculating the similarity score with BM25, k=1.5 and b=0.75 were used. After ranking the context passages, we only keep the top 100 as the input of the question-answering model.

#### Question-Answering Model

The top 100 abstracts selected by the context retriever are used as the input context passages to generate answers to the related question. A valid answer is no longer than 50 words and the end position is always behind the start position. The context passages where the question-answering model fails to generate a valid answer are discarded, and only the valid answers are used in the opinion aggregator.

#### Opinion Aggregator

The number of topics used in the BTM is 40. The range of candidate numbers of opinions (k) for k-means is from 2 to 5. For each opinion, the words with high frequency are selected as the top words manually.

## Results

### Principal Results

The results based on all CORD-19 articles, articles published since 2019, and those published since 2020 are summarized in Tables 1-3. For each question, the opinions acquired by the proposed pipeline are presented. Along with each opinion, its corresponding high-frequency words are also listed to facilitate understanding and interpretation.

**Figure 2:**
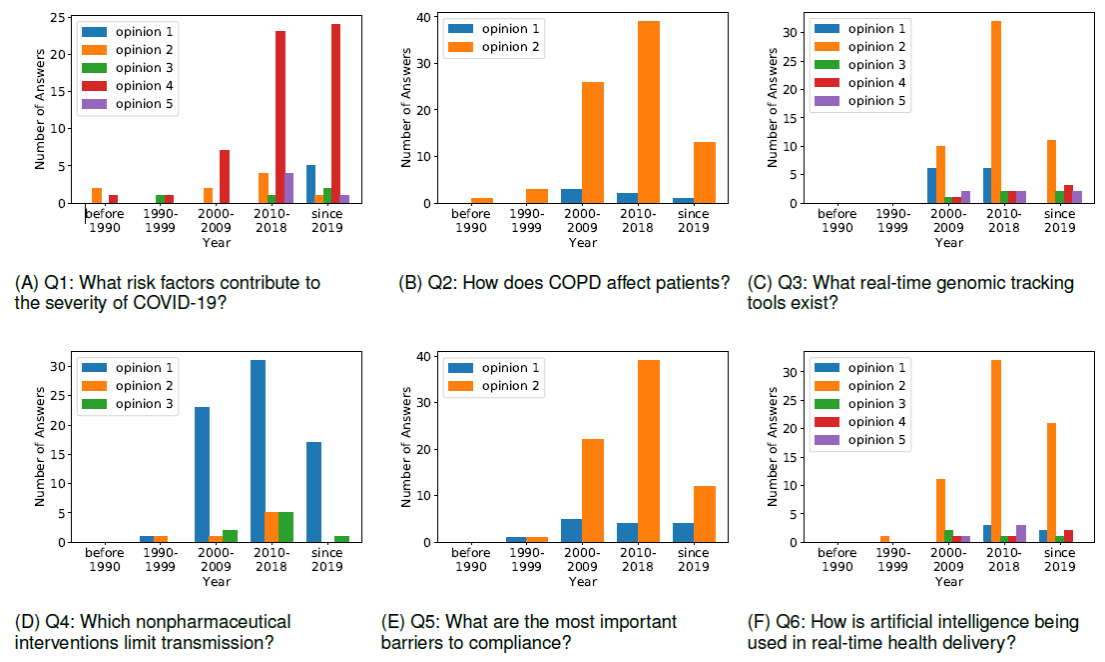
The graphs show the number of articles affiliated with each opinion during different periods for all questions. The numbers are based on all articles. The interpretation of the opinions is shown in Table 1. COPD: chronic obstructive pulmonary disease.

For the results acquired based on all CORD-19 articles, we further demonstrate the trend of research over time through the popularity of opinions. Figure 2 presents the number of articles under each opinion published before 1990, during 1990-1999, 2000-2009, and 2010-2018, and since 2019 for all questions.

**Table 1:** Opinions, the number of answers supporting every question, and the words with the highest frequency for each opinion based on all articles.

Question and opinion		Number of answers^a^	Top words in answers
**Question 1: What risk factors contribute to the severity of COVID-19?**			
	1. Transmission and spread	5	Transmission, spread
	2. Environmental factors	9	Factors, host, viral, environmental
	3. Allergy and preventive therapies	4	N/A^b^
	4. Exposure, infection, and clinical disease	56	Disease, factors, viral, clinical, age, inflammatory
	5. Interventions for modifiable risk factors	5	Risk, factors
**Question 2: How does chronic obstructive pulmonary disease affect patients?**			
	1. Substantial morbidity	6	Morbidity, mortality, hospital
	2. Exacerbations	82	Exacerbations, pulmonary, chronic, obstructive, respiratory, acute
**Question 3: What real-time genomic tracking tools exist?**			
	1. Polymerase chain reaction	12	PCR, RT, real, time, reverse
	2. Annotation tools	53	Tracking, genomic, sequencing
	3. Microarrays	5	Viral, genome
	4. Web-based tools	6	Analysis, genomic
	5. Tools to manipulate this system are growing	6	Computational, tracking
**Question 4: Which nonpharmaceutical interventions limit transmission?**			
	1. Quarantine, isolation, and social distancing	72	Quarantine, interventions, social, isolation, distancing, closure
	2. Invasive devices	7	Vaccines, antiviral
	3. Education about alcohol-based hand sanitizer, and education about hand sanitizer and face masks	8	Hand, face, masks, hygiene, washing, sanitizer, alcohol, based
**Question 5: What are the most important barriers to compliance?**			
	1. Physical barriers	14	Species, physical
	2. Perceived barriers	74	Control, lack, perceived, infection, knowledge
**Question 6: How is artificial intelligence being used in real-time health delivery?**			
	1. Text mining	5	Remote, monitoring, control
	2. Data fusion	65	Public, epidemic, decision, surveillance, detection
	3. Monitors and disseminates online information about emerging infectious diseases	4	Online, information
	4. Reactive. It is necessary to understand the complexity and interactions of integrated environmental health risks	4	Necessary
	5. Can be incorporated into any real-time polymerase chain reaction assay	4	Incorporated, RT, PCR, assay

^a^Number of answers classified to each opinion (ie, in each k-means cluster).

^b^N/A: not applicable.

**Table 2:** Opinions, the number of answers supporting every question, and the words with the highest frequency for each opinion in articles published since 2019.

Question and opinion		Number of answers^a^	Top words in answers
**Question 1: What risk factors contribute to the severity of COVID-19?**			
	1. Global spread	6	Transmission, spread
	2. Genetic and environmental factors	6	Environmental, genetic
	3. Older age and high number of comorbidities	56	Age, patients, infections, viral, diabetes, acute, hypertension, illness, older, comorbidities, respiratory, pathogens
	4. Interventions and intensity	5	Interventions, potential
**Question 2: How does chronic obstructive pulmonary disease affect patients?**			
	1. May affect the disease status	63	Disease, chronic, pulmonary, clinical, immune, virus, obstructive, cells, infections, asthma
	2. According to the severity of the disease and patients were followed up to the clinical endpoint	5	Treatment, severity, disease
	3. Treated with antibiotics	6	Higher, risk
	4. They will also be affected as ordinary citizens	6	Mortality, morbidity
**Question 3: What real-time genomic tracking tools exist?**			
	1. Benchmark databases	8	Models, multiple
	2. Real-time reverse transcription polymerase chain reaction and genomic sequencing	12	PCR, quantitative, sequencing, RT, reverse, transcription, TaqMan
	3. Close contacts	54	Analysis, tools, hosts, sequence, polymerase, genomic
	4. Extensive testing and case tracking	5	Testing
	5. Prediction tools with distinct algorithms	5	Prediction, forecasting
**Question 4: Which nonpharmaceutical interventions limit transmission?**			
	1. Protective measures and social distancing	10	Social, distancing, measures, quarantine
	2. Limit further transmission	57	Measures, epidemic, social, host, infection, travel, reduce, screening, testing, population, transmission, limit, viral, vascular, ml
	3. Continuum limit of this model is a system of delay differential equations	4	N/A^b^
	4. No spurious polymerase chain reaction ampliﬁcation occurred from the genomic RNA or DNA of other pathogens	3	PCR, ampliﬁcation, RNA
	5. Without other interventions	11	Health, public, China, implemented
**Question 5: What are the most important barriers to compliance?**			
	1. Species barriers	11	Species, ﬁtness
	2. Extracellular barriers	76	Hand, compliance, hygiene, lack, health, epithelial, practices, respirators, information, medical
**Question 6: How is artificial intelligence being used in real-time health delivery?**			
	1. Real-world study	72	Data, based, epidemic, system, model, remote, decision, information, provide, tools, health, control, detection, infectious, surveillance, CRISPR
	2. Real-time reverse transcription polymerase chain reaction was used to detect the new coronavirus in respiratory samples	9	PCR, RT, swab

^a^Number of answers classified to each opinion (ie, in each k-means cluster).

^b^N/A: not applicable.

**Table 3:** Opinions, the number of answers supporting every question, and the words with the highest frequency for each opinion in the articles published since 2020.

Question and opinion		Number of answers^a^	Top words in answers
**Question 1: What risk factors contribute to the severity of COVID-19?**			
	1. Transmission and spread	5	Transmission, spread, characteristics
	2. Nosocomial infection	58	Age, cases, patients, higher, risk, incidence, older, respiratory, diabetes, hypertension, comorbidities, severe, pneumonia, admission, acute, infection, development, lower, viral, levels, elevated, conditions
	3. Natural and social factors	7	Interventions, natural
	4. Knowledge of the confirmed case fatality risk	4	Screening, knowledge, cCFR
**Question 2: How does chronic obstructive pulmonary disease affect patients?**			
	1. It affects all age groups, including newborns and older adults	4	Increased, all, age, groups
	2. According to the severity of the disease, patients were followed up to the clinical endpoint	4	Disease, severity, followed, clinical
	3. They will also be affected as ordinary citizens	3	Affected
	4. Mixing of viral components during coinfection alters pathogenic outcomes	5	Injury, higher, function
	5. Coronavirus pneumonia	57	Mortality, clinical, changes, host, respiratory, high, growth, chronic, disease, asthma, immune, viral, reduced, cases, symptomatic, virus, effects
**Question 3: What real-time genomic tracking tools exist?**			
	1. CodSeqGen	5	Models, multiple
	2. Quantitative real-time polymerase chain reaction assays	7	PCR, rt, quantitative, assays
	3. Tools and analytical capabilities	9	Analytical, epidemiological, twitter, tracing
	4. Sequenced	5	Based
	5. Close contacts	53	Data, testing, human, analysis, reverse, methods
**Question 4: Which nonpharmaceutical interventions limit transmission?**			
	1. Social distancing and movement restrictions	11	Social, distancing, restrictions, measures, travel, movement
	2. Multidisciplinary comprehensive interventions	6	N/A^b^
	3. Screening program	5	Screening
	4. Public health intervention	12	Health, public, China, measures, implemented, reduce
	5. Limit further transmission	49	Measures, epidemic, social, pharmaceutical, infection, outbreaks, limit, population, vascular, ml, distancing, human, rate, reduce, peak, prevention, coverage, closures, testing, importation, respond
**Question 5: What are the most important barriers to compliance?**			
	1. Infectious pathogens	80	Transmission, virus, medical, protection, research, cross, practice, measures, patients, immune, respiratory, ophthalmic, infection, staff, wildlife, limited, disinfection, isolation
	2. Population movement	7	Cellular
**Question 6: How is artificial intelligence being used in real-time health delivery?**			
	1. Cesarean section	5	Monitor
	2. Modified mRNA (modRNA)	63	Data, rt, PCR, clinical, epidemic, system, model, modified
	3. A combination of repeated swab tests and computed tomography scanning	4	Repeated
	4. Methods and findings using the traditional SEIR model	5	World, model, network
	5. Drug delivery	6	Surveillance, drug, systems

^a^Number of answers classified to each opinion (ie, in each k-means cluster).

^b^N/A: not applicable.

### An Example to Illustrate the NLP Pipeline

We used the question “What are the most important barriers to compliance?” and the articles published since 2019 as an example to illustrate the proposed pipeline. First, the top relevant passages are selected by the context retriever. For example, one relevant passage selected is as follows:

Timely detection of novel coronavirus (2019-nCoV) infection cases is crucial to interrupt the spread of this virus. We assessed the required expertise and capacity for molecular detection of 2019-nCoV in specialized laboratories in 30 European Union European Economic Area (EU EEA) countries. Thirty-eight laboratories in 24 EU EEA countries had diagnostic tests available by 29 January 2020. A coverage of all EU EEA countries was expected by mid-February. Availability of primers/probes, positive controls and personnel were main implementation barriers.

Second, the question and the passage are combined as a single sequence as an input to the BERT model. According to the common BERT practice, the sequence starts with the special <CLS> token, and the question and passage are separated by the <SEP> token. The pretrained BERT question-answering model returns the start (Availability) and end locations (personnel), and the resulting answer is a fraction of the passage “Availability of primers/probes, positive controls and personnel.” The other passages returned by the BM25 context retriever were also used as input of the pretrained BERT question-answering model, and the answer in each passage is identified similarly.

Third, the answers in the passages are used as the input of the BTM model to obtain feature vectors, which are the input of the k-means clustering used to estimate the number of clusters (ie, opinions in response to the COVID-19–related questions). For the question concerning barriers to compliance, two opinions (ie, clusters) were identified. The number of answers classified to each opinion, the top words in the answers for each opinion, and some sample answers for each opinion are shown in Table S1 in Multimedia Appendix 1. The results for another question (Which nonpharmaceutical interventions limit transmission?) are shown in Table S2 in Multimedia Appendix 1. The results for all questions given articles from different periods are discussed in the following sections.

### Results Based on All CORD-19 Articles

For the first question (What risk factors contribute to the severity of COVID-19?), over half of the answers are related to exposure, infection, and the pre-existence of other clinical diseases. The distributions of opinions during different periods for question 1 are shown in Figure 2A. In the last decade, more studies are related to environmental factors and related interventions. Recently, the number of studies on transmission and spread has increased substantially, which agrees with the significantly higher transmissibility of COVID-19. Regarding the second question (How does chronic obstructive pulmonary disease affect patients?), most studies focus on the process of chronic obstructive pulmonary disease (COPD) compared to morbidity. The number of articles on this opinion has increased gradually over the years, as shown in Figure 2B.

For the third question (What real-time genomic tracking tools exist?), research about real-time genomic tracking tools for coronaviruses started to gain attention after the year 2000. Most efforts have been focused on annotation tools for genomic sequencing and tracking. The second topic frequently studied is related to polymerase chain reaction (PCR). The other three topics, which include novel tracking methods (microarrays, web-based tools, and computational tools), received relatively less attention before 2019, as shown in Figure 2C. However, they have attracted more and more attention during the COVID-19 pandemic, as demonstrated when comparing the numbers in the past year to those in the past 10 years.

For the fourth question (Which nonpharmaceutical interventions limit transmission?), three topics have been studied: (1) quarantine and social distancing, (2) vaccines, and (3) education about using hand sanitizer and face masks. Most publications focus on the first topic. More publications about topics 2 and 3 appeared in the second decade of this century than the first decade, as shown in Figure 2D.

In answering the fifth question (What are the most important barriers to compliance?), most of the studies tend to focus more on perceived barriers than physical barriers, as shown in Figure 2E. For the sixth question (How is artificial intelligence being used in real-time health delivery?), research on using artificial intelligence (AI) has attracted increasing attention since the year 2000, as shown in Figure 2F. Most of the selected publications contribute to using AI for detection, surveillance, epidemic research, and decisions. Other AI applications include remote and online monitoring and bioinformatics applications.

### Results Based on CORD-19 Articles Since 2019 and Since 2020

To better understand the studies published near the outbreak of COVID-19, we repeated the analysis using the publications that appeared since 2019 and since 2020, respectively. The results are compared to those based on all publications. Specifically, we compared the opinions of the answers and the top words among these time frames and highlighted research trends.

The recently reported high-risk factors (question 1) are more related to senior people [26,27] and pre-existing medical conditions [28], which have been reported to be important characteristics of COVID-19. Compared to previous publications about the effect of COPD (question 2) that focused on various symptoms and pre-existing conditions, recent publications have studied these together with mortality. The reason may be that patients with COVID-19 experience a wide range of disease severity (eg, asymptomatic, mild disease, severe disease, death). Furthermore, the number of deaths is substantially larger than the number of deaths from other viruses from the same family [29]. Regarding nonpharmaceutical interventions that limit transmission (question 4), the recent emphasis has been on screening and testing, given that COVID-19 is highly contagious and spreads rapidly. The barriers to compliance (question 5) have shifted from perceived barriers to the challenges of COVID-19, such as rapid transmission and the implementation of practical interventions (eg, protection, research, disinfection, and isolation). As indicated by the publications, the most noticeable increase of an AI application (question 6) is related to PCR tests.

## Discussion

### Principal Findings

To determine what the CORD-19 literature reports about COVID-19–related questions, we proposed a pipeline to select relevant passages with a BM25 context retriever, find answers by applying a BERT question-answering model, and distill the main opinions using a BTM-based opinion aggregator and k-means clustering. The results demonstrate the usefulness of the proposed method in answering COVID-19–related questions with main opinions as well as determining their popularity in the literature and capturing the trends in research about COVID-19 and other relevant strains of coronavirus in recent years. The discovered trends agree with the findings about and challenges related to COVID-19.

### Limitations

There are several limitations in our current study. First, in the current analysis, we only incorporated the abstracts of articles as the knowledge source. Considering full articles would provide more information but require more computational power. We may also need to account for multiple answers in an article. Second, for the opinion aggregator, we used a BTM topical model to obtain features for clustering. Semantical paraphrasing methods are expected to result in better representation and clustering results in future work.

### Comparison With Prior Work

Compared to several very recent studies using question-answering models for CORD-19, our study has several unique contributions. First, our proposed system can automatically aggregate the answers to a user query into a few main opinions, which can prevent users from getting overwhelmed by a large number of differing answers and help users grasp the current major opinions related to the query.

Second, compared to a large set of independent articles, the aggregated opinions provide a more robust representation of the research ideas, together with their popularity as quantified by the number of supportive articles. Third, considering COVID-19 research is still ongoing, there will likely be new ideas, discoveries, and arguments put forward in the future that will better answer questions about COVID-19. Compared to arbitrary answers generated by these recently proposed methods or developed systems, our approach shows all the existing opinions, which may be more suitable for open questions and more informative for researchers. Finally, we demonstrate comprehensive data analysis results and interpretations to provide useful insights into COVID-19 research.

### Conclusions

Compared with other methods for literature retrieval and answer generation, the opinion aggregation of our method leads to a more interpretable, robust, and comprehensive question-specific literature review. The results demonstrate the usefulness of the proposed method in answering COVID-19–related questions with main opinions and capturing the coronavirus research trends of recent years.

## Appendix

Multimedia Appendix 1 Supplementary tables.

## Abbreviations

AI: artificial intelligence
BERT: bidirectional encoder representations from transformers
BTM: biterm topic model
COPD: chronic obstructive pulmonary disease
CORD-19: COVID-19 Open Research Dataset Challenge
MERS-CoV: Middle East respiratory syndrome coronavirus
PCR: polymerase chain reaction
SARS-CoV: severe acute respiratory syndrome coronavirus
TF-IDF: term frequency–inverse document frequency
