# Supplementary material for: Revealing Opinions for COVID-19 Questions Using a Context Retriever, Opinion Aggregator, and Question-Answering Model: Model Development Study
Source: J Med Internet Res. 2021 Mar 19;23(3):e22860. doi: 10.2196/22860 (PMC7984426; doi:10.2196/22860)
Supplement: Multimedia Appendix 1 [file jmir_v23i3e22860_app1.docx]

**Multimedia Appendix 1**

Table S1. Answers and most frequent words to the questions “What are the most important barriers to compliance?” using the articles after 2020.

| Opinions | physical barriers | perceived barriers |
| --- | --- | --- |
| #answers under each opinion | 14 (16%) | 74 (84%) |
| most frequent words for each opinion | species, physical, access | control, lack, perceived, infection, knowledge |
| sample answers under each opinion | accessibility barriers;  barriers to access; | doubts about vaccine efficacy and fear of adverse reactions;  ease of use and belief in its effectiveness;  handwashing and wearing masks, gloves, and gowns |

Table S2. Answers and most frequent words to the questions “What are the most important barriers to compliance?” using the articles after 2020.

| opinions | 1.quarantine, isolation, and social distancing | 2.invasive devices | 3.education with alcohol based hand sanitizer, and education with hand sanitizer and face masks |
| --- | --- | --- | --- |
| #answers under each opinion | 72 (83%) | 7 (8%) | 8 (9%) |
| most frequent words for each opinion | quarantine interventions social isolation distancing closure | vaccines antiviral | hand face masks hygiene washing sanitizer alcohol based |
| sample answers under each opinion | voluntary quarantine;  social distancing measures;  social distancing, contact tracing, and isolation quarantine;  quarantine or other isolation measures | vaccination and antiviral medications;  antiviral therapeutics; | hand hygiene, wearing face masks, social distancing;  hand washing, alcohol based hand sanitizer use and hand washing after touching contaminated surfaces;  hand washing, face mask use, avoidance of public transport |
